# Supplementary material for: Maternal and child immune profiles are associated with neurometabolite measures of early-life neuroinflammation in children who are HIV-exposed and uninfected: a South African birth cohort
Source: Wellcome Open Res. 2026 Mar 9;10:611. Originally published 2025 Oct 31. [Version 2] doi: 10.12688/wellcomeopenres.24911.2 (PMC12982982; doi:10.12688/wellcomeopenres.24911.2)
Supplement: Peer review history

# Reviewer report: Reviewer response for version 2

**DOI:** 10.21956/wellcomeopenres.28715.r150236

**Published:** 2026-03-18

**Authors:** Ashley Harris

**Affiliations:** 1 Department of Radiology, University of Calgary, Calgary, Canada

I approve of the author's response. This will be a nice contribution to the literature.

Is the work clearly and accurately presented and does it cite the current literature?

Yes

Are all the source data underlying the results available to ensure full reproducibility?

Yes

Is the study design appropriate and is the work technically sound?

Yes

Are the conclusions drawn adequately supported by the results?

Yes

Are sufficient details of methods and analysis provided to allow replication by others?

Yes

Reviewer Expertise:

NA

I confirm that I have read this submission and believe that I have an appropriate level of expertise to confirm that it is of an acceptable scientific standard.

# Reviewer report: Reviewer response for version 2

**DOI:** 10.21956/wellcomeopenres.28715.r150508

**Published:** 2026-03-17

**Authors:** Richard Glashoff

**Affiliations:** 1 Division of Medical Microbiology & Immunology, Stellenbosch University, Cape Town, South Africa

This is a well executed and presented body of research. I am reviewing a revised version - having been through initial peer review already. I think the initial manuscript was overly confident in stating what the findings mean. This has been toned down significantly. It is always difficult to precisely define the meaning of associations in this type of study - particularly where the markers themselves are associated with multiple things in different contexts. As it now stands the revised version presents some key immune-neurometabolite associations in a well-defined mother child HEU cohort.

 The key findings were the association between IL-5 and IL-8 in mothers with myo-inositol ratios (MIR) in midline parietal grey and right parietal white matter regions. Other associations (e.g. MMP-9 and MIR in midline parietal grey matter in 2 year-old children and lack of IL-13 and glutamate ratios in midline parietal grey matter of HEU which was seen in uninfected control children) were also noted.

 The study relies on extensive statistical analysis. It appears that the statistics are very stringent with BH correction. The authors have highlighted in certain instances where relationships were significant prior to BH adjustment - which us useful in a study of this nature.

 The data are well presented and generally easy to follow. The conclusions are cautiously stated and the findings are novel and worthy of publication.

 I have a few minor concerns / questions / suggestions. The choice of inflammatory markers is not justified. Was it simply based on an available multiplex panel or were the markers chosen for any specific reason?

 The study utilizes serum (as opposed to plasma). Was this a choice? The clotting process can alter relative concentrations of certain cytokines and other markers - so we (and many others) prefer plasma. It shouldn't be a major issue here if everything is done on the same source material. The only issue is that absolute values may differ with other studies which use plasma.

 There is some controversy regarding the use of serum markers in CNS association studies. The peripheral blood and CNS appear very separate compartments with minimal overlap. This should perhaps be alluded to here. Obviously it is not practical and also ethically questionable to obtain CSF from all participants, however the relationhip between blood markers and measures of brain function can be complicated by virtue of the fact that the blood does not reflect the CSF. This also begs the question whether the associations observed here would "hold" in a CSF study. This is not a major sticking point - but should perhaps be mentioned.

 Is this the first study of this kind on a mother child HEU cohort? It wasn't clear whether all the findings were novel or whether certain associations had been seen before elsewhere. This could be better emphasized.

 The IL-5 and IL-8 maternal association with HEU parietal grey and white matter regions, respectively - needs more discussion. Especially IL-5 (IL-8 to some extent is expanded upon). The IL-5 finding is unexpected. It is normally associated with allergic inflammation and eosinophil function. There is no indication of whether mothers were allergy sufferers - was this assessed? IL-5 may impact epithelial / barrier function and may indirectly impact in BBB integrity. This should be alluded to. IL-8 is a classical neutrophil chemokine and often elevated in inflammatory scenarios. It too may impact on BBB function. I felt this mechanistic aspect was not discussed enough (and is really exciting for a cytokine immunologist).

 Finally, was analysis of any stratified data with regard to ART initiation before or during pregnancy performed? It would be interesting to see whether the extent of in utero exposure has on markers and CNS associations. This also stratifications also applies to stratification for nadir CD4 or peak viral load.

Is the work clearly and accurately presented and does it cite the current literature?

Yes

If applicable, is the statistical analysis and its interpretation appropriate?

I cannot comment. A qualified statistician is required.

Are all the source data underlying the results available to ensure full reproducibility?

Yes

Is the study design appropriate and is the work technically sound?

Yes

Are the conclusions drawn adequately supported by the results?

Yes

Are sufficient details of methods and analysis provided to allow replication by others?

Yes

Reviewer Expertise:

HIV immunology, cytokine biology, innate immunity

I confirm that I have read this submission and believe that I have an appropriate level of expertise to confirm that it is of an acceptable scientific standard.

# Reviewer report: Reviewer response for version 2

**DOI:** 10.21956/wellcomeopenres.28715.r150234

**Published:** 2026-03-12

**Authors:** Jaime H Vera

**Affiliations:** 1 Brighton and Sussex Medical School, Brighton, UK

I am satisfied with the work done by the authors addressing the comments

Is the work clearly and accurately presented and does it cite the current literature?

Yes

If applicable, is the statistical analysis and its interpretation appropriate?

Yes

Are all the source data underlying the results available to ensure full reproducibility?

Yes

Is the study design appropriate and is the work technically sound?

Yes

Are the conclusions drawn adequately supported by the results?

Yes

Are sufficient details of methods and analysis provided to allow replication by others?

Yes

Reviewer Expertise:

HIV and brain health

I confirm that I have read this submission and believe that I have an appropriate level of expertise to confirm that it is of an acceptable scientific standard.

# Reviewer report: Reviewer response for version 2

**DOI:** 10.21956/wellcomeopenres.28715.r150235

**Published:** 2026-03-12

**Authors:** Kim Cecil

**Affiliations:** 1 Cincinnati Children's Hospital Medical Center, Cincinnati, USA

I appreciate the authors thoughtful addressing of my concerns. I have no further recommendations.

Is the work clearly and accurately presented and does it cite the current literature?

Yes

If applicable, is the statistical analysis and its interpretation appropriate?

Partly

Are all the source data underlying the results available to ensure full reproducibility?

Partly

Is the study design appropriate and is the work technically sound?

Partly

Are the conclusions drawn adequately supported by the results?

Partly

Are sufficient details of methods and analysis provided to allow replication by others?

Yes

Reviewer Expertise:

Magnetic resonance spectroscopy

I confirm that I have read this submission and believe that I have an appropriate level of expertise to confirm that it is of an acceptable scientific standard.

# Reviewer report: Reviewer response for version 1

**DOI:** 10.21956/wellcomeopenres.27441.r139380

**Published:** 2025-12-24

**Authors:** Ashley Harris

**Affiliations:** 1 Department of Radiology, University of Calgary, Calgary, Canada

This study examines data from the Drakenstien Child Health Study to gain new insight about children who are HIV-exposed and uninfected as across development they are at higher risk of neurodevelopmental delays. The two groups are HEU and HU – denoting HIV-exposed-uninfected and HIV unexposed). This study examined blood serum markers in these children and their mothers (mothers during pregnancy, children at 6weeks and 2year) as well as single voxel MRS data in the children at 2 years (though abstract says 23 years – I think that must be a typo). The analysis include examining the differences and trajectories in these blood biomarkers.

 I think this is a well executed study with an impressive amount of data. I have several comments but they are all minor and intended to provide clarity as I expect there audience will be broad (clinicians and researchers with or without expertise in child development, blood serum analyses and spectroscopy).  

 Reviewing the previous reviewer comments – I do not see how the choline and NAA exclusions were adjusted. There is no comment about these data being excluded so perhaps they have just been included for completeness? Please clarify.

 Other comments:

 Introduction

- Is there a citation for the DCHS study as a whole? Would be nice to include
- Final paragraph could be more clear as I’m confused about what is novel and what the sampling is – specifically have already published the MRS data (correct?) so the addition is longitudinal blood data or is there longitudinal MRS data as well?

Methods

- Reports of participants – This is confusing as it is partial information. It is all in the results so I would just state that the data is collected as apart of the DCHS study and then report the data that is used in the results as this is more appropriate and it is clearly written/understood in the results section right now.
- HIV status section doesn’t seem to fit well in the sociodemographic methods – perhaps would be best in own section
- Never stated what sociodemographic information was collected
- What is GM-CSF? I assume IL-XX is interleukin-XX, I don’t know what INF-gamma is either (I do recognize these are standard assay to those who do these blood biomarkers but have targeted a diverse audience with this manuscript), similarly fill name of MMP-9, NAGL, YKL-40 is likely appropriate and the brief descriptions are appreciated.
- Would be beneficial to quantify relative to water and perform full quantification (see Near et al 2020) if have unsuppressed water data as creatine may be changing, if you don’t have water data for quantification, I think this should be acknowledged.
- Units for FWHM missing and the metabolite used or the SNR and FWHM for quality assessment

Results

- The description of the participants in the results is much clearer – suggest just reporting the metrics acquired in the methods
- I cannot figure out how to get the supplemental figures so have not examined. From the text they appear to be appropriate.
- Figure 1 – very interesting results - the font on the axes (even in the downloaded ppt) is too small. Can see zoomed in on pdf but recommended fixing
- If I understand, a limited correction for multiple comparisons was performed – only corrected for number that were significant in the primary analysis, not corrected for all comparisons performed.
  - Given serum markers didn’t change but have different results in the 6 week and 2 year data – this raises a suspicion that should be acknowledged but I don’t see as a fatal flaw

Discussion

- Discussion is appropriate but largely a summary of the results with a little context. I think it would be compelling to interpret and speculate on the data more – does this give direction for clinicians now? Should actions be taken to consider the immune profiles and perhaps brain development trajectories for these children?

Is the work clearly and accurately presented and does it cite the current literature?

Yes

Are all the source data underlying the results available to ensure full reproducibility?

Yes

Is the study design appropriate and is the work technically sound?

Yes

Are the conclusions drawn adequately supported by the results?

Yes

Are sufficient details of methods and analysis provided to allow replication by others?

Yes

Reviewer Expertise:

spectroscopy, pediatric imaging.

I confirm that I have read this submission and believe that I have an appropriate level of expertise to confirm that it is of an acceptable scientific standard, however I have significant reservations, as outlined above.

# Response

**Published:** 2026-02-12

**Authors:** Cesc Bertran Cobo

**Affiliations:** University of Cape Town Department of Psychiatry and Mental Health, Observatory, Western Cape, South Africa

***Reviewer – **Overall Impression: "I think this is a well executed study with an impressive amount of data. I have several comments but they are all minor and intended to provide clarity as I expect there audience will be broad (clinicians and researchers with or without expertise in child development, blood serum analyses and spectroscopy).  Reviewing the previous reviewer comments – I do not see how the choline and NAA exclusions were adjusted. There is no comment about these data being excluded so perhaps they have just been included for completeness? Please clarify.*

***"*** **Response** – We thank the reviewer for the thoughtful and constructive feedback and for emphasising clarity for a broad audience spanning clinicians and researchers with varied expertise. Regarding choline and NAA adjustments, we apologize for the confusion caused by our earlier wording. As outlined in the Reviewer #1 response, choline and NAA were not excluded from statistical testing; they were analysed in the same models as other metabolites with voxel tissue fractions included a priori as covariates, highlighting their sensitivity to tissue composition in adjusted models.

***Reviewer **– Other comments:*

*1.    Introduction*

*1.1.    Is there a citation for the DCHS study as a whole? Would be nice to include* Response – We have added citations for the DCHS cohort overview at first mention in the Introduction section (line 25), in addition to the existing protocol citations in Methods. *Reviewer – 1.2.    Final paragraph could be more clear as I’m confused about what is novel and what the sampling is – specifically have already published the MRS data (correct?) so the addition is longitudinal blood data or is there longitudinal MRS data as well?*

**Response** – Thank you, we have reviewed the Introduction paragraph for clarity. Wording now reads as follows (line 55, Introduction section, last paragraph): “Our study aimed to address this gap by investigating maternal and child peripheral blood immune profiles from pregnancy through birth and early childhood, and their associations with brain metabolite levels at age 2–3 years, using existing data from the DCHS cohort of South African children.”

***Reviewer – 2.**    Methods*

*2.1.    Reports of participants – This is confusing as it is partial information. It is all in the results so I would just state that the data is collected as apart of the DCHS study and then report the data that is used in the results as this is more appropriate and it is clearly written/understood in the results section right now.*

**Response** – Thank you. We have retained a concise Methods description (with detailed protocol references) and added a signposting sentence indicating that final analytic sample sizes by timepoint and modality are reported in the Results and Supplementary Figure 1. Wording now reads as follows (line 88, Methods section, end of 2nd paragraph): “Final sample sizes contributing to each analysis are reported in the Results and Supplementary Figure 1.” ***Reviewer – 2.2**.    HIV status section doesn’t seem to fit well in the sociodemographic methods – perhaps would be best in own section*

**Response **– Thank you. We agree and have created a new sub-section within the Methods section, called “Maternal and child HIV status” (line 88, in Methods section).

***Reviewer – 2.3**.    Never stated what sociodemographic information was collected*

**Response – **We have added a brief sentence listing the main sociodemographic and psychosocial domains collected (income, education/employment, relationship status, smoking/alcohol, depression, infant feeding and growth), including references to the detailed DCHS protocol publications. Wording now reads as follows (line 102, Methods section, Sociodemographic data collection sub-section): “Variables included household income, maternal education and employment, relationship status, smoking and alcohol use during pregnancy, depressive symptoms, birth outcomes, infant feeding, and child growth indicators.” ***Reviewer – 2.4.**    What is GM-CSF? I assume IL-XX is interleukin-XX, I don’t know what INF-gamma is either (I do recognize these are standard assay to those who do these blood biomarkers but have targeted a diverse audience with this manuscript), similarly fill name of MMP-9, NAGL, YKL-40 is likely appropriate and the brief descriptions are appreciated.*

**Response **–We agree and have included IFN-γ notation and defining GM-CSF, IFN-γ, and TNFα at first use. We also note that a comprehensive marker table (names/abbreviations and relevant literature) is provided in the Supplementary Files, and we have enquired with the editors about displaying this table in the main text, as we believe this addition provides clarification for broader audiences. Wording now reads as follows (line 114, Methods section, Immune assays sub-section): “Concentrations of granulocyte–macrophage colony-stimulating factor (GM-CSF), interferon gamma INF(IFN-γ), interleukins IL-1β, IL-2, IL-5, IL-6, IL-7, IL-8 (CXCL8), tumour necrosis factor alpha (TNF-α), IL-4, IL-10, IL-12p70, and IL-13 were measured using a Milliplex ® Luminex 13-plex kit (#HSTCMAG28SPMX13; Merck) according to the manufacturer’s instructions.”

***Reviewer – 2.5**.    Would be beneficial to quantify relative to water and perform full quantification (see Near et al 2020) if have unsuppressed water data as creatine may be changing, if you don’t have water data for quantification, I think this should be acknowledged.*

**Response **– Unsuppressed water reference scans were not available, therefore, we report metabolite ratios to total creatine and not absolute concentrations. Wording now reads as follows (line 150, Methods section, MRS data processing sub-section): “Unsuppressed water reference scans were not available, therefore, we do not report neurometabolite absolute concentrations.

**”** ***Reviewer **– 2.6.    Units for FWHM missing and the metabolite used or the SNR and FWHM for quality assessment*

**Response – **We clarified the FWHM units (ppm) and the QC approach. Wording now reads as follows (line 150, Methods section, MRS data processing sub-section): “Spectral quality was visually inspected and assessed using signal-to-noise ratio (SNR) and full width at half maximum (FWHM, in ppm) given by LCModel. Spectra with FWHM values greater than 0.08 or SNR values lower than 10 were considered of low quality and therefore excluded.” ***Reviewer – 3.**    Results*

*3.1.    The description of the participants in the results is much clearer – suggest just reporting the metrics acquired in the methods*

**Response –** Thank you, we proceeded as suggested. Wording now reads as follows (line 102, Methods section, Sociodemographic data collection sub-section): “Variables included household income, maternal education and employment, relationship status, smoking and alcohol use during pregnancy, depressive symptoms, birth outcomes, infant feeding, and child growth indicators.

**”** ***Reviewer – 3.2**.    I cannot figure out how to get the supplemental figures so have not examined. From the text they appear to be appropriate.*

**Response **– Please see line 495 (Data availability section, Extended data sub-section). We mention there that Supplementary Figures are contained within the “Supplementary Files (PDF)” deposited in the OSF project linked by DOI.

***Reviewer – 3.3.**    Figure 1 – very interesting results - the font on the axes (even in the downloaded ppt) is too small. Can see zoomed in on pdf but recommended fixing*

**Response –** Thank you. We have corrected Figure 1 by increasing axis font sizes for readability and have submitted it to the Editorial Team to have it replaced in the main text. ***Reviewer – 3.4**.    If I understand, a limited correction for multiple comparisons was performed – only corrected for number that were significant in the primary analysis, not corrected for all comparisons performed.*

**Response –** We clarified that Benjamini–Hochberg correction was applied across all tests within each analysis set, irrespective of unadjusted p-values. Wording now reads as follows (line 195, Methods section, Statistical analysis sub-section, last paragraph): “Results were considered significant at a p-value threshold of < 0.05 (two-tailed). BH correction was applied across all tests within each analysis set.

**”** ***Reviewer – 3.5**.    Given serum markers didn’t change but have different results in the 6 week and 2 year data – this raises a suspicion that should be acknowledged but I don’t see as a fatal flaw*

**Response – **Previous studies, as well as our findings, demonstrate increases in cytokine levels during the first two years of life, with subsequent stabilisation, consistent with immune system maturation in early life.

- Ray JE, Dobbs KR, Ogolla SO, et al. Clinical and Immunological Outcomes of HIV-Exposed Uninfected and HIV-Unexposed Uninfected Children in the First 24 Months of Life in Western Kenya. BMC Infectious Diseases (2023) (1):156. doi: 10.1186/s12879-024-09051-3. 
- Miyamoto M, Gouvêa AFTB, Ono E, et al. Immune development in HIV-exposed uninfected children born to HIV-infected women. Rev Inst Med Trop Sao Paulo (2017) 59:e30. doi: 10.1590/S1678-9946201759030. 

***Reviewer – 4.**    Discussion*

*4.1.    Discussion is appropriate but largely a summary of the results with a little context. I think it would be compelling to interpret and speculate on the data more – does this give direction for clinicians now? Should actions be taken to consider the immune profiles and perhaps brain development trajectories for these children?*

**Response **– We strengthened the Discussion by adding a paragraph on clinical insights and next steps, emphasizing that findings are exploratory, but that they support the importance of optimising maternal health during pregnancy. Wording now reads as follows (line 466, Discussion section, last paragraph): “In conclusion, this study suggests that maternal HIV status is associated with selective alterations in maternal and child immune profiles, and with exposure-specific associations between a subset of maternal/child serum markers and child neurometabolite ratios at 2–3 years. These findings provide preliminary evidence that perinatal HIV exposure may be followed by altered early-life neuroimmune coupling, although associations are marker-specific and require replication. Clinically, our results reinforce the importance of optimising maternal HIV health during pregnancy, with early ART initiation and monitoring and viral suppression to minimise immune activation, as well as prevention and management of inflammatory comorbidities. Future studies should confirm our findings in larger cohorts, integrate more detailed social and environmental determinants, and link perinatal immune profiles to longitudinal neurodevelopmental trajectories to clarify mechanisms and identify modifiable pathways that support optimal development in children who are HEU.”

# Reviewer report: Reviewer response for version 1

**DOI:** 10.21956/wellcomeopenres.27441.r138485

**Published:** 2025-12-11

**Authors:** Jaime H Vera

**Affiliations:** 1 Brighton and Sussex Medical School, Brighton, UK

This is an important and well-designed study. The paper presents unique longitudinal immune data paired with MRS outcomes in a South African HEU cohort something rarely available. However, several aspects need clarification to avoid overstating conclusions and to ensure the interpretation matches the strength of the results.

 1. Contextualisation of prior literature

 The introduction summarises well the inconsistent findings across geographical regions. I suggest explicitly highlighting that these discrepancies may also reflect differences in social determinants of health, environmental exposures, and co-infections, which can shape immune maturation. This helps the reader understand that immune differences in HEU children are not solely attributable to HIV exposure.

 2. Over-interpretation of immune brain associations

 Only a subset of immune markers (e.g., IL-5, IL-8, MMP-9, NGAL) showed significant associations. Many initial differences did not survive multiple-comparison correction. Statements implying widespread associations should be softened to reflect that the findings relate to specific markers, not broad immune dysregulation.

 3. Limited adjustment for social determinants

 The manuscript does not adjust for social determinants of health despite clear group differences. These factors, which strongly influence inflammation and neurodevelopment, likely contribute to observed differences. The authors should acknowledge this in the limitations.

 4. Interpretation of non-significant infant/child immune differences

 Most differences in infant and child serum markers disappear after correction. This should be stated clearly, emphasising that the data are more consistent with normalisation of markers over time rather than persistent dysregulation.

 5. Mediation analysis interpretation

 The mediation models did not show evidence that serum markers explain the effect of maternal HIV on neurometabolites. This may reflect small sample size, weak group differences, unmeasured social determinants, or that immune pathways may not be the primary mechanism.

 6. Final conclusion too strong

 The conclusion should use more cautious language (“suggests”, “indicates”, “provides preliminary evidence”) rather than “demonstrates”, given the exploratory nature of the findings and potential confounding.

 This study offers valuable insight into maternal and child immune profiles and early neurometabolic patterns in HEU children. Analyses are rigorous, but interpretation should be more cautious. Clarifying the influence of social determinants, emphasising that only specific markers show associations, and softening conclusions will strengthen the manuscript and ensure alignment with the statistical evidence.

Is the work clearly and accurately presented and does it cite the current literature?

Yes

If applicable, is the statistical analysis and its interpretation appropriate?

Yes

Are all the source data underlying the results available to ensure full reproducibility?

Yes

Is the study design appropriate and is the work technically sound?

Yes

Are the conclusions drawn adequately supported by the results?

Yes

Are sufficient details of methods and analysis provided to allow replication by others?

Yes

Reviewer Expertise:

HIV and brain health

I confirm that I have read this submission and believe that I have an appropriate level of expertise to confirm that it is of an acceptable scientific standard, however I have significant reservations, as outlined above.

# Response

**Published:** 2026-02-12

**Authors:** Cesc Bertran Cobo

**Affiliations:** University of Cape Town Department of Psychiatry and Mental Health, Observatory, Western Cape, South Africa

***Reviewer **– Overall Impression: "This is an important and well-designed study. The paper presents unique longitudinal immune data paired with MRS outcomes in a South African HEU cohort something rarely available. However, several aspects need clarification to avoid overstating conclusions and to ensure the interpretation matches the strength of the results."* **Response **– We thank the reviewer for their careful reading of the manuscript and for the constructive suggestions to better align our interpretation with the strength of the evidence. We have clarified the role of contextual factors and use more cautious language to strengthen the paper.  

***Reviewer **– 1. Contextualisation of prior literature: "The introduction summarises well the inconsistent findings across geographical regions. I suggest explicitly highlighting that these discrepancies may also reflect differences in social determinants of health, environmental exposures, and co-infections, which can shape immune maturation. This helps the reader understand that immune differences in HEU children are not solely attributable to HIV exposure."*  

**Response **– We agree and have expanded the Introduction to explicitly note that heterogeneity in immune findings in children who are HEU may reflect social determinants of health, environmental exposures, and co-infections that may shape early immune maturation.   We have added text highlighting these contextual influences and include supporting references: “These inconsistencies may reflect differences in regional exposure to co-infections, social determinants of health, and early-life environmental factors that influence immune maturation ^A,B^. They also highlight the need for further research to elucidate how HIV exposure affects immune and neurodevelopmental trajectories in children across different social and geographical contexts.”   A. Kuhlman KR, Horn SR, Chiang JJ, Bower JE. Early life adversity exposure and circulating markers of inflammation in children and adolescents: A systematic review and meta-analysis. Brain Behav Immun. 2020;86:30-42. doi: 10.1016/j.bbi.2019.04.028.   B. Gilman SE, Hornig M, Ghassabian A, et al. Socioeconomic disadvantage, gestational immune activity, and neurodevelopment in early childhood. Proc Natl Acad Sci USA. 2017;114(26):6728-6733. doi: 10.1073/pnas.1617698114.  

***Reviewer** – 2. Over-interpretation of immune brain associations. "Only a subset of immune markers (e.g., IL-5, IL-8, MMP-9, NGAL) showed significant associations. Many initial differences did not survive multiple-comparison correction. Statements implying widespread associations should be softened to reflect that the findings relate to specific markers, not broad immune dysregulation."*  

**Response **– Thank you. We have revised the Discussion to emphasise that associations were identified for specific serum markers and that many differences did not survive multiple comparison correction. We softened wording that could imply widespread immune dysregulation and reframed the findings as selective neuroimmune associations that differ by HIV exposure group.   Examples of rewording include:

- Abstract conclusions: “In this South African cohort, HIV exposure-specific associations were observed between certain mother and child immune markers and child neurometabolite ratios at 2–3 years.”
- Line 381 (Discussion, 3rd paragraph): “Together, these findings support our hypothesis that specific maternal and child immune markers are associated with early-life elevated myo-inositol in the parietal white matter of children who are HEU.”
- Lines 386–390 (Discussion, 3rd paragraph): “Myo-inositol is clinically recognized as a marker of neuroinflammation and gliosis, and it has been linked to cognitive impairment in children living with HIV. Thus, increases in myo-inositol alterations in children who are HEU may potentially result from maternal immune dysregulation during pregnancy. IL-8 is a plausible candidate in this context, given its role as a pro-inflammatory chemokine in perinatal immune activation and, if dysregulated, its potential negative effects on brain maturation and neurodevelopment.
- Line 465 (Discussion, last paragraph): “In conclusion, this study suggests that maternal HIV status is associated with selective distinct alterations in maternal and child immune profiles, and with exposure-specific associations between a subset of maternal/child serum markers and child neurometabolite ratios at 2–3 years.”

 
***Reviewer **– 3. Limited adjustment for social determinants: "The manuscript does not adjust for social determinants of health despite clear group differences. These factors, which strongly influence inflammation and neurodevelopment, likely contribute to observed differences. The authors should acknowledge this in the limitations."*  

**Response **– We clarified that key socioeconomic variables were comparable between HEU and HU groups in this subset, and that we conducted sensitivity analyses for measured variables that did differ between groups. We agree that residual confounding remains possible and have expanded the limitations to acknowledge that we could not account for additional factors that may influence immune development and neurometabolite associations.   Wording now reads as follows (line 445, Discussion section, Strengths and limitations paragraph): “While socioeconomic status was similar across children who were HEU and HU, maternal age at delivery, as well as rates of depression and alcohol use during pregnancy differed between groups. Reassuringly, our results held after correcting for these variables in sensitivity analyses, although other unmeasured factors may contribute to neuroimmune variability.”  

***Reviewer **– 4. Interpretation of non-significant infant/child immune differences: "Most differences in infant and child serum markers disappear after correction. This should be stated clearly, emphasising that the data are more consistent with normalisation of markers over time rather than persistent dysregulation."*  

**Response **– We agree and edited the Discussion to more explicitly state that, after correction, group differences were primarily observed during pregnancy and that infant/child differences were attenuated, which is more consistent with partial normalisation of peripheral markers over time than persistent dysregulation across early life.   Wording now reads as follows (line 374, Discussion section, end of 2nd paragraph): “Despite this, our longitudinal analysis from six weeks to two years did not reveal significant differences in serum marker trajectories between HEU and HU children. This may be consistent with partial normalisation rather than persistent differences of peripheral markers over time in early childhood.”  

***Reviewer **– 5. Mediation analysis interpretation: "The mediation models did not show evidence that serum markers explain the effect of maternal HIV on neurometabolites. This may reflect small sample size, weak group differences, unmeasured social determinants, or that immune pathways may not be the primary mechanism."*  

**Response **– Thank you, we agree and have reviewed the mediation paragraph in the Discussion to consider the interpretation of the mediation results. We have added potential explanations including limited power in complete-case mediation models, small indirect effects, attenuation of child marker differences after correction, residual confounding by unmeasured social/environmental factors, and others. We consider that immune pathways may not be primary mechanisms but equally, the results do not exclude immune mechanisms but suggest that no single measured marker explained the maternal HIV–neurometabolite association in isolation.   Wording now reads as follows (line 413, Discussion section, 5th paragraph): “Our mediation analyses did not identify any individual serum marker that mediated the association between maternal HIV status and child neurometabolite ratios at 2–3 years. This null result should be interpreted cautiously given limited power to detect indirect effects in our complete-case with paired immune and MRS data. Moreover, group differences in several serum markers were modest and further attenuated after multiple-comparison correction, reducing power to detect mediation by any single marker. Residual confounding by social and environmental determinants, as well as biological pathways (including antiretroviral exposure and co-infections), may also play a role. Taken together, these findings do not rule out immune mechanisms but suggest that no single measured marker explains the maternal HIV–neurometabolite association in isolation.”  

***Reviewer **– 6. Final conclusion too strong: "The conclusion should use more cautious language (“suggests”, “indicates”, “provides preliminary evidence”) rather than “demonstrates”, given the exploratory nature of the findings and potential confounding."* **Response** – We have adopted more cautious language as suggested and emphasise the exploratory nature of the findings and the need for larger, longitudinal studies integrating neurodevelopmental outcomes and contextual determinants.

 Wording now reads as follows (line 465, Discussion, last paragraph): “In conclusion, this study suggests that maternal HIV status is associated with selective distinct alterations in maternal and child immune profiles, and with exposure-specific associations between a subset of maternal/child serum markers and child neurometabolite ratios at 2–3 years.”

# Reviewer report: Reviewer response for version 1

**DOI:** 10.21956/wellcomeopenres.27441.r138490

**Published:** 2025-11-25

**Authors:** Kim Cecil

**Affiliations:** 1 Cincinnati Children's Hospital Medical Center, Cincinnati, USA

Summary:  HIV-exposed and uninfected (HEU) children are at risk of neurodevelopment delays from prenatal immune dysregulation due potentially to exaggerated neuroinflammatory responses. However, no studies have longitudinally examined if changes in serum marker levels throughout childhood for HIV-exposed and uninfected (HEU) children are related to brain metabolite profiles in early life. Using data from the Drakenstein Child Health Study, the investigators collected maternal serum samples at 26-28 weeks gestation and children at 6 weeks and 2 years of age. They also previously collected proton magnetic resonance spectroscopy from three brain regions of a subset of the cohort. The investigators examined cross-sectional associations between immune markers and neurometabolite ratios with linear models adjusting for age at scan, sex, and voxel tissue composition and controlling for false discovery rate.

 Overall Impression: The manuscript was well-organized, well-written and flowed very well. There is one major concern in the analyses and a few minor issues.

 Major concern: On page 8 within results sub-section, the cross-sectional associations between serum markers and brain metabolite ratios, the authors state they excluded choline ratios to creatine from all three voxels. They also state they excluded N-acetyl aspartate ratios to creatine in the left parietal white matter. I interpreted that this was due to variations in voxel placement across participants during the study acquisition as the authors stated "influence of tissue composition" and "suboptimal tissue composition." From my experience, this exclusion procedure is very troublesome. I am sure that many studies have suffered from this error in acquisition and have not accounted for it. It likely contributes to inconsistencies in associations in the literature. While it has been reported in the literature the differences in gray and white matter have a known effect on choline levels, I am not confident that glutamate and myo-inositol are not also impacted. I would need the authors to address this exclusion practice as valid. Alternatively, they come up with another approach for addressing this limitation. It is noted that the original publication of the MRS dataset accounted for gray matter within white matter voxel in a different fashion.

 All sections of the manuscript (abstract, introduction, methods, results and discussion) included relevant material in a succinct fashion. The design and participant information could be readily understood. The discussion provided appropriate context for the findings. I think the glutamate and myo-inositol findings are likely valid, however, the excluding of NAA/Cr and Cho/Cr from the neurometabolite outcomes is not a valid post-processing practice from my perspective. 

 Minor items: 1. Abstract results. The reader does not yet know mixed-effects models will be used, so the sentence "In children without HIV exposure, higher maternal anti-inflammatory IL-13 was associated with higher glutamate ratios in midline parietal grey matter (β=0.42, p<0.0001), and this association was significantly modified by maternal HIV (interaction β=−0.41, p=0.038)" is confusing with children without HIV and modified by maternal HIV.

 2. Abstract Conclusions: ratios at 23 years is missing the hyphen: it should be 2-3 years.

 3. BH (Benjamini-Hochberg) is not defined at first use in the statistical analysis section of the methods, but later in the results in group differences in maternal and child serum marker concentrations.

Is the work clearly and accurately presented and does it cite the current literature?

Yes

If applicable, is the statistical analysis and its interpretation appropriate?

Partly

Are all the source data underlying the results available to ensure full reproducibility?

Partly

Is the study design appropriate and is the work technically sound?

Partly

Are the conclusions drawn adequately supported by the results?

Partly

Are sufficient details of methods and analysis provided to allow replication by others?

Yes

Reviewer Expertise:

Magnetic resonance spectroscopy

I confirm that I have read this submission and believe that I have an appropriate level of expertise to state that I do not consider it to be of an acceptable scientific standard, for reasons outlined above.

# Response

**Published:** 2026-02-12

**Authors:** Cesc Bertran Cobo

**Affiliations:** University of Cape Town Department of Psychiatry and Mental Health, Observatory, Western Cape, South Africa

We thank the reviewer for the careful evaluation of our manuscript and for highlighting both strengths and areas needing clarification. We have addressed the queries regarding voxel tissue composition and neurometabolite ratio interpretation and updated the manuscript accordingly. ***Reviewer – Major concern**:* *"On page 8 within results sub-section, the cross-sectional associations between serum markers and brain metabolite ratios, the authors state they excluded choline ratios to creatine from all three voxels. They also state they excluded N-acetyl aspartate ratios to creatine in the left parietal white matter. I interpreted that this was due to variations in voxel placement across participants during the study acquisition as the authors stated "influence of tissue composition" and "suboptimal tissue composition." From my experience, this exclusion procedure is very troublesome. I am sure that many studies have suffered from this error in acquisition and have not accounted for it. It likely contributes to inconsistencies in associations in the literature. While it has been reported in the literature the differences in gray and white matter have a known effect on choline levels, I am not confident that glutamate and myo-inositol are not also impacted. I would need the authors to address this exclusion practice as valid. Alternatively, they come up with another approach for addressing this limitation. It is noted that the original publication of the MRS dataset accounted for gray matter within white matter voxel in a different fashion.* *All sections of the manuscript (abstract, introduction, methods, results and discussion) included relevant material in a succinct fashion. The design and participant information could be readily understood. The discussion provided appropriate context for the findings. I think the glutamate and myo-inositol findings are likely valid, however, the excluding of NAA/Cr and Cho/Cr from the neurometabolite outcomes is not a valid post-processing practice from my perspective."* **Response – **Thank you for raising this. We agree that our wording in the Results section could be interpreted as post-hoc exclusion, and we apologise for the lack of clarity. We would like to clarify that Cho/Cr and NAA/Cr were not removed from the dataset or omitted from statistical testing. Because the proportion of white matter in the white matter voxels was ≈52%, we included voxel tissue fractions a priori as covariates in all regression models to reduce partial-volume confounding. In these adjusted models, Cho/Cr across all voxels and NAA/Cr in the left parietal white matter voxel showed significant associations with tissue fraction (p<0.05), which might limit biological interpretability and increase the risk of residual partial-volume effects for these outcomes. By contrast, the main outcomes highlighted in the manuscript (Glu/Cr, Ins/Cr, and NAA/Cr in parietal grey matter and right parietal white matter) were robust to tissue-fraction adjustment. It is also important to note that most unadjusted associations involving Cho/Cr or and left parietal white matter NAA/Cr did not survive Benjamini–Hochberg correction for multiple comparisons, or lost significance after covariate adjustment. The only association that remained statistically significant after all steps was the association between maternal IL-10 during pregnancy and left parietal white matter NAA/Cr in HIV-unexposed children. However, given the strong dependence of this outcome on tissue fraction in the adjusted model, we believe it should be interpreted cautiously. We have revised the manuscript text to avoid any implication of post-hoc exclusion, and we now report this IL-10 association in the Results section with appropriate caution. Applied changes include:

- Lines 182–185 (Methods section, Statistical analysis sub-section, end of 3rd paragraph): Explanation reworded to “Given that the percentage of white matter in white matter voxels was ≈52% in our study, tissue composition was also included a priori in the adjusted analysis. This was intended to reduce partial volume confounding when testing associations between serum markers and neurometabolites.”
- Line 275 (Results section, Cross-sectional associations sub-section, 1st paragraph): The following text has been deleted to avoid misunderstanding: “Adjusted analyses revealed a significant influence of tissue composition on total choline ratios across all parietal voxels, therefore, this metabolite was excluded from all analyses. N-acetyl-aspartate ratios in the left parietal white matter were also excluded from our analyses due to suboptimal tissue composition.”
- Lines 324–325 (Results section, Cross-sectional associations sub-section, Maternal serum markers sub-sub-section, 3rd paragraph): The following text was added: “Maternal IL-10 during pregnancy was associated with N-acetyl-aspartate ratios in the left parietal white matter of children who were HU (β=0.30, p=0.0451) but not HEU.”
- Line 454 (Discussion section, Strengths and limitations paragraph): The following text was added: “Third, although we adjusted for voxel tissue composition, N-acetyl-aspartate ratios in the left parietal white matter voxel were sensitive to tissue fraction, limiting biological interpretability of serum associations for these outcomes in our study. Importantly, key findings for myo-inositol and glutamate ratios were robust in adjusted models, reducing concern that the primary results were artefacts of voxel mixture.”

For transparency, all unadjusted and adjusted regression outputs are provided in our OSF Statistical Analysis Report, available via the referenced OSF repository.

***Reviewer – Minor items:*** *"1. Abstract results. The reader does not yet know mixed-effects models will be used, so the sentence "In children without HIV exposure, higher maternal anti-inflammatory IL-13 was associated with higher glutamate ratios in midline parietal grey matter (β=0.42, p<0.0001), and this association was significantly modified by maternal HIV (interaction β=−0.41, p=0.038)" is confusing with children without HIV and modified by maternal HIV."* **Response **– Thank you. We have revised the abstract Results sentence to explicitly describe this as a difference in association by maternal HIV status, rather than phrasing it as a statement that applies only to HU children. The revised sentence in the abstract now reads: “Maternal IL-13 was positively associated with glutamate ratios in the midline parietal grey matter of HIV-unexposed peers (β=0.42, p<0.0001), with no association in children who are HEU”. *"2. Abstract Conclusions: ratios at 23 years is missing the hyphen: it should be 2-3 years."* **Response **– Corrected to “2–3 years”. *"3. BH (Benjamini-Hochberg) is not defined at first use in the statistical analysis section of the methods, but later in the results in group differences in maternal and child serum marker concentrations."* Response – Corrected by defining Benjamini–Hochberg (BH) at first mention in the Statistical analysis section (line 172 in the main text).
